# SARS-CoV-2 infection induces epigenetic changes in the LTR69 subfamily of endogenous retroviruses

**DOI:** 10.1186/s13100-023-00299-1

**Published:** 2023-09-04

**Authors:** Ankit Arora, Jan Eric Kolberg, Smitha Srinivasachar Badarinarayan, Natalia Savytska, Daksha Munot, Martin Müller, Veronika Krchlíková, Daniel Sauter, Vikas Bansal

**Affiliations:** 1 Institute for Stem Cell Science and Regenerative Medicine, Bengaluru, India; 2 Institute for Medical Virology and Epidemiology of Viral Diseases, University Hospital Tübingen, Tübingen, Germany; 3 Department of Molecular Biology and Genetics, Cornell University, Ithaca, NY 14853 USA; 4 German Center for Neurodegenerative Diseases (DZNE), Tübingen, Germany

**Keywords:** SARS-CoV-2, Transposable elements, Endogenous retroviruses, Solo-LTRs, LTR69

## Abstract

Accumulating evidence suggests that endogenous retroviruses (ERVs) play an important role in the host response to infection and the development of disease. By analyzing ChIP-sequencing data sets, we show that SARS-CoV-2 infection induces H3K27 acetylation of several loci within the LTR69 subfamily of ERVs. Using functional assays, we identified one SARS-CoV-2-activated LTR69 locus, termed Dup69, which exhibits regulatory activity and is responsive to the transcription factors IRF3 and p65/RELA. LTR69_Dup69 is located about 500 bp upstream of a long non-coding RNA gene (ENSG00000289418) and within the *PTPRN2* gene encoding a diabetes-associated autoantigen. Both ENSG00000289418 and *PTPRN2* showed a significant increase in expression upon SARS-CoV-2 infection. Thus, our study sheds light on the interplay of exogenous with endogenous viruses and helps to understand how ERVs regulate gene expression during infection.

**Supplementary Information:**

The online version contains supplementary material available at 10.1186/s13100-023-00299-1.

## Background

Severe acute respiratory syndrome coronavirus 2 (SARS-CoV-2), the causative agent of the COVID-19 pandemic, has caused unprecedented global health and socioeconomic impacts. With billions of infections and millions of reported deaths worldwide, there is a pressing need to better understand the complex interplay of SARS-CoV-2 with infected host cells and the pathogenesis of the disease.

Recent studies have suggested that repetitive DNA sequences known as transposable elements (TEs) play an essential role in the host response to viral infection and the development of disease. For instance, some TEs are capable of regulating the expression of antiviral factors and other host proteins through their activity as enhancers or promoters [1, 2]. Furthermore, TE-derived nucleic acids may be sensed by cellular pattern recognition receptors and thereby amplify innate sensing cascades and the induction of Interferon-mediated immune responses [3]. In line with a potential role in the outcome of viral infections, viruses such as the Human Immunodeficiency Virus (HIV), Human Cytomegalovirus (HCMV) or Influenza A Virus (IAV) trigger the activation of transposable elements that are otherwise silenced [2, 4–6].

Here, we leverage publicly available transcriptome and chromatin datasets of infected cell lines to decipher the impact of SARS-CoV-2 on the TE expression profiles of virus-infected or -exposed cells. Several studies have reported an induction of HERV-K [7–9], HERV-W [10, 11] or HERV-L [12–16] upon SARS-CoV-2 infection. In line with this, we found that SARS-CoV-2 infection results in an enrichment of transcription-associated H3K27 acetylation marks in a particular subset of human endogenous retroviruses (HERVs), so-called LTR69 repeats. These long terminal repeats (LTRs) represent solo-LTRs of the ERV-L family of endogenous retroviruses. In contrast to previous studies, we also performed mechanistic analyses and identified a SARS-CoV-2-responsive LTR69 repeat that exerts regulatory activity and is activated by IRF3 and p65/RELA, two transcription factors that are activated upon sensing of viral RNA.

## Results and discussion

**Fig. 1:**
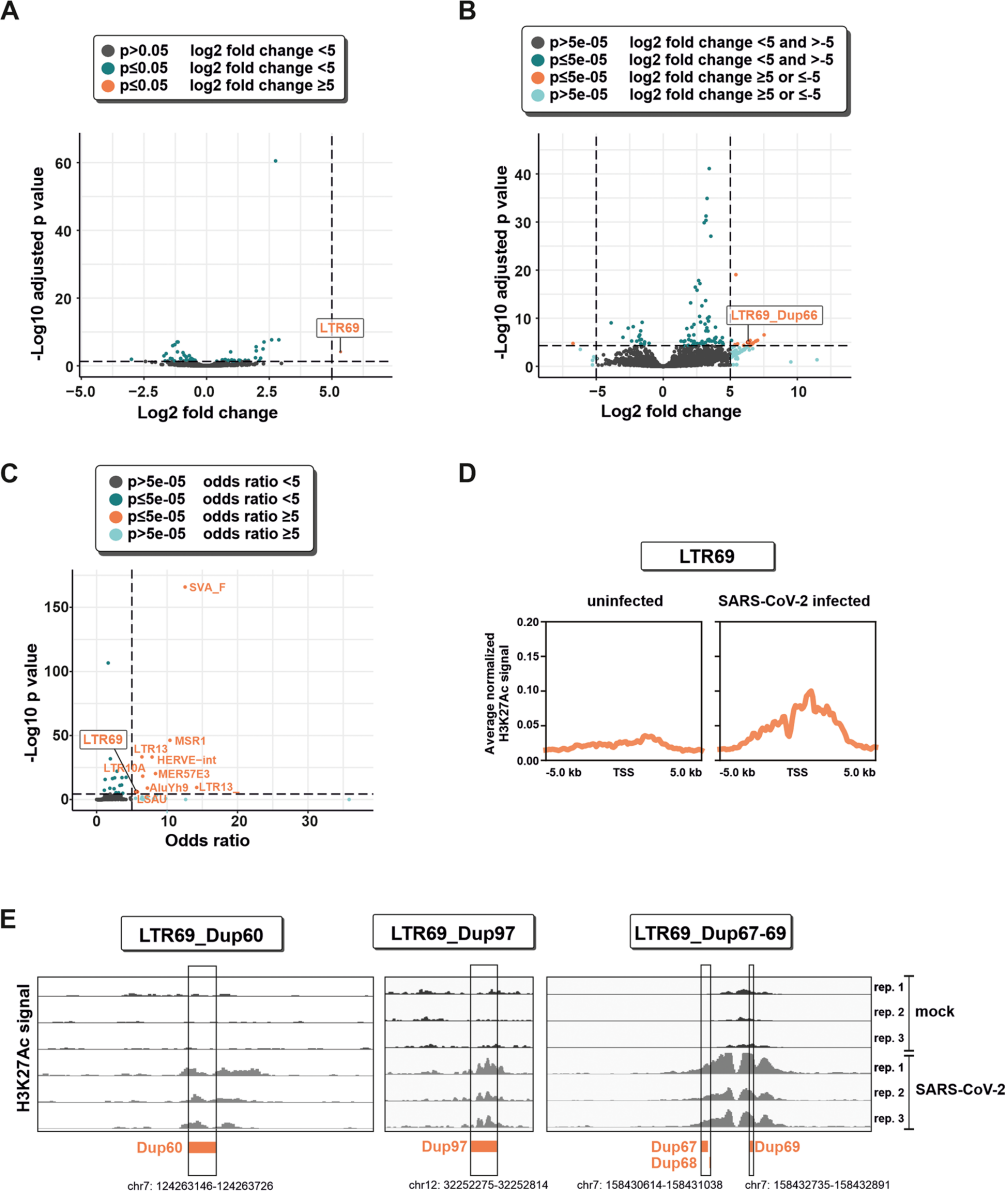
Activation of LTR69 repeats upon SARS-CoV-2 infection. **A** Volcano plot illustrating differential expression of transposable element (TEs) subfamilies in SARS-CoV-2-infected vs. uninfected Calu-3 cells (GSE147507). Dashed lines represent cutoffs of a log_2_ fold change of 5 and an adjusted *P* value of 0.05. **B** Volcano plot illustrating differential expression of individual TE loci in SARS-CoV-2-infected vs. uninfected Calu-3 cells (GSE147507). Dashed lines represent cutoffs of a log_2_ fold change of 5 and an adjusted *P* value of 5e-05. **C** Enrichment of H3K27Ac peaks in individual TE subfamilies. Dashed lines represent cutoffs of an odds ratio of 5 and a *P* value of 5e-05. **D** The average H3K27ac signal profile of LTR69 loci around the transcription start site (TSS) is shown. ChIP-seq data were obtained in SARS-CoV-2 infected (24 h, MOI 0.5) and uninfected A549-ACE2 cells. **E** Integrative Genomics Viewer (IGV) snapshots of exemplary H3K27ac peaks on individual LTR69 loci (hg38) in A549-ACE2 cells

To determine the effect of SARS-CoV-2 infection on the activity of TEs, we analyzed publicly available poly(A)-enriched mRNA-seq and ChIP-seq data. First, we took advantage of a data set from SARS-CoV-2-infected and uninfected Calu-3 cells to identify differentially expressed TEs [17]. Calu-3 cells are a human lung cell line that is susceptible to SARS-CoV-2 infection in vitro and represents the natural target cells of the virus. Using TElocal, we found one HERV subfamily, LTR69 (Log_2_ FC = 5.35 and adjusted *P*-value = 7.26e-5), to be significantly up-regulated upon SARS-CoV-2 infection (Fig. 1A and Table S1). Since LTR69 repeats represent solo-LTRs of ERV-L, these results partially support previous studies [12–16], which showed an up-regulation of ERV-L members upon SARS-CoV-2 infection. However, analysis at the single locus level revealed that only a single LTR69 repeat, Dup66, was significantly up-regulated upon SARS-CoV-2 infection (Fig. 1B and Table S2). This particular locus is located in an intron of *ZC3HAV1*, which was also significantly up-regulated (Log_2_ FC = 2.40 and adjusted *P*-value = 8.7e-99, not shown). This gene is known to be induced upon IFN stimulation and encodes the zinc-finger antiviral protein (ZAP) that restricts SARS-CoV-2 and other viral pathogens [18]. These findings strongly suggest that the activation of LTR69_Dup66 is the result of read-through transcription and responsible for the observed induction of LTR69 and the family level.

Notably, TEs can have functional consequences without being transcribed. For example, many TE-derived elements act as enhancers regulating cellular gene expression [1]. One well-recognized epigenetic mark of active enhancers is the acetylation of lysine 27 in histone H3 (H3K27Ac). To dissect the enhancer activity of TEs in the presence and absence of SARS-CoV-2, we therefore analyzed publicly available chromatin immunoprecipitation sequencing data (ChIP-seq) of H3K27Ac in A549-ACE2 cells. Using GIGGLE [19], an enrichment analysis was conducted to identify TE families that exhibit an overrepresentation of H3K27Ac peaks in infected vs. uninfected samples. This approach revealed that LTR69 displayed a significant enrichment of H3K27ac peaks (*P*-value = 6.7e-07, odds ratio = 5.6). Apart from LTR69, we have identified nine additional TE families (SVA_F, MSR1, LTR13, HERVE-int, MER57E3, LTR10A, AluYh9, LTR13_, and LSAU) out of 1180 that displayed significant associations with H3K27ac peaks (*P*-value < 0.00004 i.e. Bonferroni adjustment 0.05/1180 and odds ratio > 5) (Fig. 1C and Table S3). The transcription start site (TSS) profile plot across all LTR69 loci (*n* = 147) revealed an enrichment of H3K27Ac marks upon SARS-CoV-2 infection in comparison to uninfected cells (Fig. 1D). We focused our further analyses on individual LTR69 loci that showed at least one significant H3K27Ac peak identified by the MACS2 peak calling algorithm. There were 12 unique peaks of H3K27Ac on 15 LTR69 loci upon SARS-CoV-2 infection (Table S4). Exemplary peak signals are displayed as Integrative Genomics Viewer (IGV) screenshots in Fig. 1E.

**Fig. 2:**
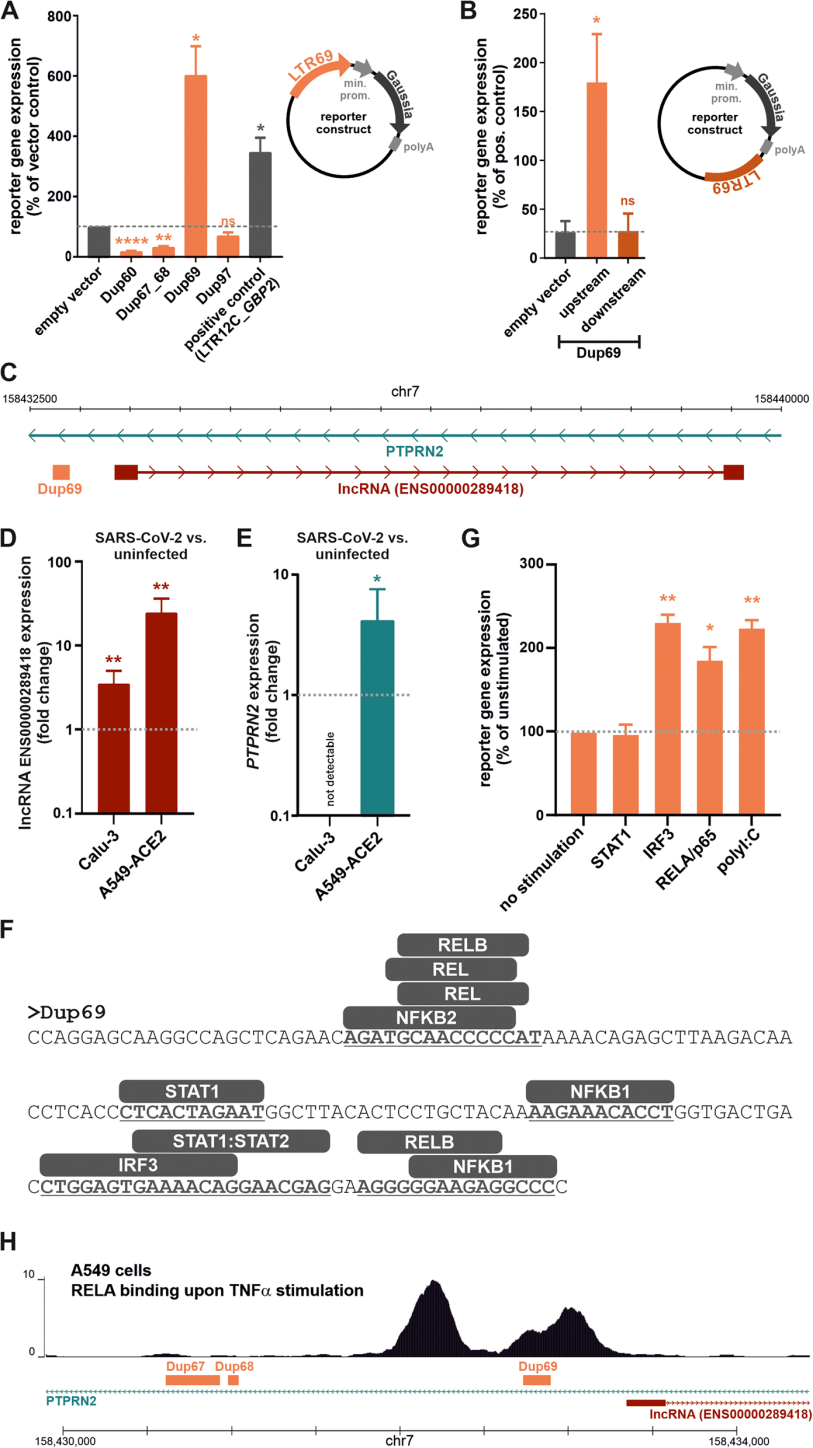
Regulatory activity of SARS-CoV-2 induced LTR69_Dup69. **A** LTR69 repeats (orange) were inserted into enhancer reporter vectors expressing *Gaussia* luciferase (black) under the control of a minimal promoter (grey). HEK293T cells were co-transfected with the indicated reporter vectors expressing *Gaussia* luciferase and a control vector expressing firefly luciferase for normalization. A previously described LTR12C repeat with known enhancer activity served as positive control. Two days post transfection, reporter luciferase activity was determined and normalized to the activity of the control luciferase. Mean values of three to four independent experiments, each performed in triplicates are shown. Error bars indicate SEM (* *p* < 0.05, ** *p* < 0.01, **** *p* < 0.0001). **B** LTR69_Dup69 was also inserted downstream of the *Gaussia* reporter gene. Reporter gene expression was determined as described in (**A**). Mean values of three to seven independent experiments, each performed in triplicates are shown. Error bars indicate SEM (* *p* < 0.05). **C** Integrative Genomics Viewer (IGV) snapshot (hg38) illustrating the localization of LTR69_Dup69 (orange) within an intron of the *PTPRN2* gene (green) and adjacent to the ENS00000289418 gene (red). **D**, **E** Expression of (**D**) ENS00000289418 and (**E**) *PTPRN2* in SARS-CoV-2-infected vs. uninfected Calu-3 and A549-ACE2 cells. Cells were infected with an MOI of 2 and harvested 24 h post infection. Mean values of two to three independent experiments ± SEM are shown. **F** Nucleotide sequence of LTR69_Dup69. The presence of putative binding sites for STAT1, NF-κB subunits and IRF3 is highlighted in bold and underlined. **G** HEK293T cells were co-transfected with the LTR69_Dup69 reporter vector expressing *Gaussia* luciferase, a control vector expressing firefly luciferase for normalization and increasing amounts of the indicated stimuli. Two days post transfection, reporter luciferase activity was determined and normalized to the activity of the control luciferase. Mean values of three independent experiments, each performed in triplicates are shown. Error bars indicate SEM (* *p* < 0.05, ** *p* < 0.01, ns not significant). **H** ChIP-seq data of TNF-α-stimulated A549 cells [20] illustrating the enrichment of RELA binding within and adjacent to LTR69_Dup69

To test whether some of the SARS-CoV-2-activated LTR69 repeats exert regulatory effects, we tested them for potential enhancer activities. We selected five representative candidates (loci names defined in the annotation file as Dup60, Dup67, Dup68, Dup69, Dup97) (Table S4) and inserted them into enhancer reporter vectors. These plasmids express a *Gaussia* luciferase reporter gene under the control of a minimal promoter, whose activity may be increased by upstream enhancer elements (Fig. 2A). Dup67 and Dup68 were inserted together into the same vector as they are located in close proximity in the genome and just separated by 54 nucleotides (Fig. 1E). A previously characterized LTR12C element located upstream of the *GBP2* gene served as positive control [2]. As expected, LTR12C_*GBP2* increased *Gaussia* luciferase expression compared to the vector control lacking an LTR repeat (Fig. 2A). A similar enhancing effect was observed for Dup69, whereas the remaining LTR69 elements had no significant modulatory effect or even decreased reporter gene expression. Unexpectedly, LTR69_Dup69 showed no enhancing effect when inserted downstream of the reporter gene (Fig. 2B). This may suggest that LTR69_Dup69 acts as a promoter rather than an enhancer. In contrast to previously characterized LTR promoters [2], however, analysis of RNA-seq data sets revealed no evidence for transcription initiation within Dup69 or any chimeric fusion transcripts involving LTR69_Dup69. Alternatively, the observation that LTR69_Dup69 only increases reporter gene expression when inserted upstream of the minimal promoter may be explained by positional effects that are required for its enhancing activity.

We focused our further analyses on LTR69_Dup69 and hypothesized that this locus might regulate the expression of adjacent genes. Inspection of the respective gene locus revealed that Dup69 is located in an intron of *PTPRN2*, about 500 nucleotides upstream of ENSG00000289418 (Fig. 2C). While *PTPRN2* codes for a tyrosine phosphatase receptor that serves as a major autoantigen in type 1 diabetes [21], ENSG00000289418 encodes a long non-coding RNA. We therefore analyzed the expression of both genes in SARS-CoV-2 infected versus uninfected lung cells via RT-qPCR. Intriguingly, expression of the lncRNA increased about 3.6- and 25.2-fold in Calu-3 and A549-ACE2, cells respectively (Fig. 2D). Furthermore, expression of *PTPRN2* increased on average 4.1-fold upon SARS-CoV-2 infection in A549-ACE2 cells, while *PTPRN2* mRNA was not detectable in Calu-3 cells (Fig. 2E). Interestingly, Sharif-Askari and colleagues also observed an up-regulation of *PTPRN2* in whole blood of COVID-19 patients [22]. *PTPRN2* expression was also significantly up-regulated (Log_2_ FC = 1.67, *P*-value = 0.04) in the RNA-seq data from A549-ACE2 cells (Table S5) corresponding to the H3K27ac study described above [23]. Although a causal link remains to be demonstrated, it is tempting to speculate that changes in the expression of ENSG00000289418 and/or *PTPRN2* are mediated by the regulatory activity of the LTR69-Dup69 repeat. To elucidate the mechanisms that may underlie the activation of LTR69_Dup69 upon SARS-CoV-2 infection, we screened its nucleotide sequence for binding sites of transcription factors that are known to be activated in infected cells. Using JASPAR [24], we identified putative binding sites for NF-κB subunits (NFKB1, NFKB2, Rel), IRF3 and STAT1 (Fig. 2F). Intriguingly, LTR69 mediated enhancement of reporter gene expression could be further boosted by p65/RELA and a constitutively active mutant of IRF3, but not STAT1 (Fig. 2G). In line with an activation of IRF3 and NF-κB upon innate sensing, the synthetic dsRNA analog polyI:C also significantly increased the activity of LTR69_Dup69 (Fig. 2G). Furthermore, we investigated the Cistrome Data Browser [25] that contains RELA ChIP-seq data of TNF-alpha-stimulated A549 cells previously published by Raskatov and colleagues [20]. In line with our finding that LTR69_Dup69 is responsive to RELA, these data sets revealed that RELA is enriched within and in close proximity of LTR69_Dup69 (Fig. 2H). Notably, however, SARS-CoV-2 infection failed to increase LTR69_Dup69 driven reporter gene expression in transfected A549-ACE2 cells (Figure S1). One possible explanation is the efficient suppression of immune activation by several SARS-CoV-2 proteins that prevents activation of IRF3 and NF-κB in infected cells [26]. Furthermore, while SARS-CoV-2 infection increases H3K27 acetylation of the endogenous LTR69_Dup69 locus (Fig. 1E), the extrachromosomal LTR69_Dup69 plasmid does most likely not reflect the physiological chromatin status of this solo-LTR and may explain the lack of responsiveness in this experimental setup. Together, our findings demonstrate that SARS-CoV-2 infection results in increased H3K27 acetylation of an LTR69 repeat that is responsive to p65/RELA and IRF3 and may potentially regulate the expression of adjacent genes.

Nevertheless, we would like to point out that this study is subject to limitations. Although we analyzed and compared multiple datasets, we acknowledge that the respective sample sizes are relatively small. In the future, it would be crucial to integrate and replicate the findings in other datasets, including cell-type specific data at both transcription and chromatin layers. We would also like to point out that although expression *PTPRN2* was elevated upon SARS-CoV-2 infection, it only reached relatively low mRNA levels. The extent of change in expression certainly depends on several factors such as the infected cell type, virus strain, multiplicity of infection (MOI) and time point post-infection. Moreover, computational analysis of TEs faces significant challenges due to a high false discovery rate [27]. Therefore, the utilization of long-read RNA-seq will be important to improve both, locus-specific quantification and the analysis of chimeric transcripts. While previous studies had already analyzed the effect of SARS-CoV-2 infection on TE activity, they have used different bioinformatics tools. For example, Marston and colleagues [15] used Telescope, which analyzes full-length transposable elements. Importantly, they also observed an induction of specific ERVs in SARS-CoV-2 infected or exposed cells, including ERV-L repeats.

Future studies will shed light on the downstream effects of TE activation on the virus and its host. It will be important to identify causal links between the activity of specific regulatory TEs (e.g. LTR69 repeats) and potential cellular target genes (e.g. *PTPRN2*). In addition to LTR69, it would be intriguing to explore the involvement of the nine additional TE families that show an enrichment of H3K27Ac marks upon SARS-CoV-2 infection. Furthermore, it will be interesting to investigate whether different viral infections trigger similar transcription patterns or epigenetic changes of TEs, indicative of a broader role of transposable elements in infection and immunity.

## Conclusions

In this short report, we confirm the differential expression and activation of specific mobile genetic elements in response to SARS-CoV-2 infection. In particular, we demonstrate that one of the SARS-CoV-2-induced LTR69 loci, LTR69_Dup69, exhibits regulatory activity and is responsive to the transcription factors p65/RELA and IRF3. LTR69_Dup69 is located about 500 bp upstream of a long non-coding RNA gene, ENSG00000289418, whose expression is also increased upon SARS-CoV-2 infection. At the same time, LTR69_Dup69 is located within an intron of the *PTPRN2* gene, which is also up-regulated upon SARS-CoV-2 infection and encodes for an autoantigen involved in type 1 diabetes. While further work is required, our study identifies LTR69 repeats as transposable elements that are epigenetically modified in SARS-CoV-2 infected cells and may modulate host gene expression and thus contribute to the outcome of SARS-CoV-2 infection.

## Methods

### RNA-seq data collection

The RNA-seq datasets analyzed in this study were downloaded from the GEO database with accession numbers GSE147507 (SRX8089276 to SRX8089281) for Calu-3 and GSE162619 (GSM4955401 to GSM4955406) for A549-ACE2.

### Transcriptome quantification and differential expression analysis

RNA-seq quality control and trimming were performed using fastp [v0.20.1] [28] followed by aggregation of the QC report data in MultiQC [v1.9] [29]. STAR [v2.7.5a] [30] was used for mapping using GRCh38 as a reference genome (the additional flags –outFilterMultimapNmax 100 –winAnchorMultimapNmax 10 –outSAMtype BAM Unsorted –outFilterMismatchNmax 999 –outFilterMismatchNoverLmax 0.1). This was followed by gene and transposon locus level quantification using TElocal [v.0.1.0] [31] (https://github.com/mhammell-laboratory/TElocal) in Unique Mode as per our previously tested optimal performance parameters [27] using gencode [v29] [32] and LNCipedia [v5.2] [33] for gene annotation and GRCh38 repeatmasker (downloaded from the TElocal developer repository) for TE loci annotation. TE subfamily level quantification was achieved using count aggregation across all duplicates per subfamily. Genes and transposable elements were analyzed for differential expression separately, using DESeq2 [v1.30] [34]. TE loci were visualized for inspection using IGV [2.11.1] [35].

### ChIP-seq analysis

ChIP-seq data were downloaded from GSE167528 [23]. Raw ChIP-seq reads in fastq format were subjected to quality control using FastQC [v0.11.9] [36]. Quality controlled reads were subjected to removal of adapter sequences and quality filtering using fastp [v0.20.1]. The filtered reads were mapped to the human (hg38) reference genome using bowtie2 [v2.3.0] [37]. Peak calling on mapped reads was performed using MACS2 [v2.1.1] [38], merged infected (GSM5106727, GSM5106728, GSM5106729) BAM file vs merged uninfected (GSM5106721, GSM5106722, GSM5106723) BAM file. In addition, bigwig signals and matrix computation were performed for each condition using deepTools [v2.5.2] [39]. GIGGLE [v0.6.3] was used to perform enrichment analysis with default parameters (https://github.com/ryanlayer/giggle). H3K27ac infection-specific peaks (infected vs. uninfected) were used as index and each family was searched against the index (Table S3).

### Infection of Calu-3 and A549-ACE2 cells with SARS-CoV-2 for RT-qPCR

One day before infection, A549-ACE2 (80,000 cells/well) or Calu-3 (200,000 cells/well) were seeded into a 48-well plate. Cells were infected with SARS-CoV-2 B.1 at an MOI = 2 and incubated at 37°C for 24 h.

### RT-qPCR

RNA was isolated from infected cells 24 h p.i. using Qiagen RNeasy Mini Kit (Cat. #74106). gDNA was eliminated using Invitrogen DNA-*free*™ DNase Treatment & Removal (Cat. #AM1906). cDNA was synthesized using Applied Biosystems High-Capacity cDNA Reverse Transcription Kit with RNase Inhibitor (Cat. #4374966). qPCR was performed using New England Biolabs Luna® Universal qPCR Master Mix (Cat. #M3003L). All steps were performed according to the manufacturer’s instructions. The following primers were used:

**Table:** 

ENSG00000289418 Fwd:	5′ GAA GTT TAC AGG CAA AAG CTG C 3′
ENSG00000289418 Rev:	5′ AAC CCA GTG CCA GGA ATG AA 3′
GAPDH Fwd:	5′ GAG TCC ACT GGC GTC TTC A 3′
GAPDH Rev:	5′ GGG GTG CTA AGC AGT TGG T 3′

The following primer-probes were used for the PTPRN2 qPCR:

- PTPRN2 FAM ThermoFischerScientific (Cat. #4448892; AssayID: Hs00243067_m1)
- GAPDH VIC ThermoFisherScientific (Cat. #4448489).

All reactions were performed in duplicates, and GAPDH was used to normalize RNA expression across all samples. Raw RT-qPCR data is provided in Table S6.

### Reporter plasmids

To generate promoter reporter vectors, LTR69 and LTR12C loci were synthesized (GenScript) and inserted into the pGLuc Mini-TK 2 *Gaussia* luciferase enhancer reporter plasmid (NEB) via KpnI/SacI restriction sites, upstream of the minimal promoter, as previously described [2]. In one case, LTR69_Dup69 was inserted downstream of the *Gaussia* luciferase gene using the Takara In-Fusion® HD Cloning Kit according to manufacturer’s instructions.

### Cell culture

Human embryonic kidney 293 T (HEK293T) cells and A549-ACE2 cells were cultured in Dulbecco’s modified Eagle medium (DMEM) containing 10% heat-inactivated fetal calf serum (FCS), 2 mM glutamine, 100 μg/ml streptomycin and 100 units/ml penicillin. HEK293T cells were tested for mycoplasma contamination every three months. Only mycoplasma negative cells were used for this study. Calu-3 were cultured in Dulbecco’s modified Eagle medium (DMEM) containing 10% heat-inactivated fetal calf serum (FCS) plus 2 mM glutamine, 100 μg/ml streptomycin and 100 units/ml penicillin. Medium was changed daily.

### Prediction of transcription factor binding sites

Putative binding sites for NF-κB subunits, IRF3 and STAT1 were predicted using JASPAR 2022 [24]. *Homo sapiens* was selected as species, and the relative profile score threshold was set to 70%. The ten sequence motifs with the highest relative scores (0.74–0.80) are shown in Fig. 2F.

### Enhancer reporter assay

HEK293T cells were seeded (30,000 cells/well) in poly-L-lysine coated 96-well tissue culture plates. After 24 h, cells were transfected with a combination of expression vectors expressing *Gaussia* luciferase under the control of a minimal herpes simplex virus (HSV) thymidine kinase promoter (25 ng), either alone or upstream of a LTR12C or LTR69 locus, as well as a pTAL firefly luciferase plasmid (50 ng) as a normalization control and polyI:C (2,000 ng) or an expression plasmid for p65 (100 ng), STAT1 (100 ng) or a constitutively active mutant of IRF3 (1,000 ng). After 24 h, supernatants were harvested, and cells were lysed in 40 µl 1 × Passive Lysis Buffer (Promega). *Gaussia* luciferase activity in the supernatants was measured by addition of Coelenterazine (PJK Biotech) and firefly luciferase activity was measured in the cells using the Luciferase Assay System (Promega) according to the manufacturer’s instructions.

A549-ACE2 cells were seeded (22,000 cells/well) in 96-well tissue culture plates. After 24 h, cells were transfected with a combination of vectors expressing Gaussia luciferase under the control of a minimal herpes simplex virus (HSV) thymidine kinase promoter (240 ng), either alone or downstream of a LTR12C or LTR69 locus, as well as a pTAL firefly luciferase plasmid (480 ng) for normalization. Cells were transfected using the Invitrogen™ Lipofectamine 2000 Transfection Reagent (Cat. #11668019) according to the manufacturer’s instructions. 6 h post transfection, the medium was replaced and cells were infected with SARS-CoV-2 B.1 at an MOI = 0.2 or MOI = 2 and incubated at 37°C for 24 h. Supernatants were harvested and inactivated using 1% Triton™ X-100. Cell lysis and luciferase measurements were performed as described above.

### Supplementary Information

**Additional file 1.**

**Additional file 2.**

**Additional file 3.**

**Additional file 4.**

**Additional file 5.**

**Additional file 6.**

**Additional file 7: Figure S1.** LTR69-driven reporter gene expression in SARS-CoV-2-infected A549-ACE2 cells.

## Data Availability

All data generated or analyzed during this study are included in this published article [and its supplementary information files].
