# Supplementary material for: SARS-CoV-2 infection induces epigenetic changes in the LTR69 subfamily of endogenous retroviruses
Source: Mob DNA. 2023 Sep 4;14:11. doi: 10.1186/s13100-023-00299-1 (PMC10476400; doi:10.1186/s13100-023-00299-1)
Supplement: Supplementary file 7 — Additional file 7: Figure S1. LTR69-driven reporter gene expression in SARS-CoV-2-infected A549-ACE2 cells. [file 13100_2023_299_MOESM7_ESM.pdf]

FIGURE S1

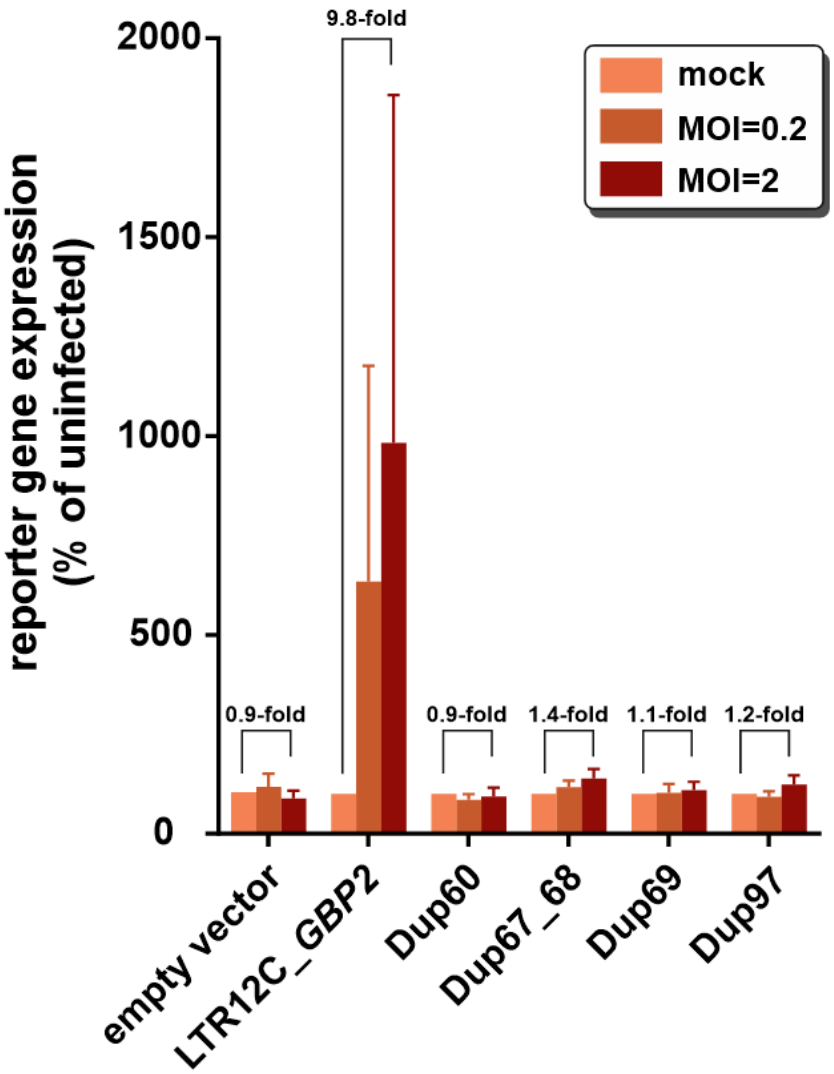

Figure S1: LTR69-driven reporter gene expression in SARS-CoV-2-infected A549-ACE2 cells. A549-ACE2 cells were co-transfected with the indicated reporter vectors expressing *Gaussia* luciferase and a control vector expressing firefly luciferase for normalization. A previously described LTR12C repeat with known enhancer activity served as positive control. 6 h post transfection, cells were infected with SARS-CoV-2 at an MOI of 0.2 or 2. 24 h later, reporter luciferase activity was determined and normalized to the activity of the control luciferase. Mean values of three to five independent experiments, each performed in triplicates are shown. Error bars indicate SEM.
